# Zygomatic Implants for Rehabilitation of the Atrophic Maxilla: Clinical Indications, Outcomes, Complications, and Evidence Gaps—A Comprehensive Review

**DOI:** 10.3390/medicina62071277

**Published:** 2026-07-02

**Authors:** Alfonso Acerra, Alessandro Santurro, Cristian Coraini, Andrea Enrico Borgonovo, Angelo Aliberti, Francesco Giordano

**Affiliations:** 1 Department of Medicine, Surgery and Dentistry “Scuola Medica Salernitana”, University of Salerno, 84081 Baronissi, Italy; asanturro@unisa.it (A.S.); frgiordano@unisa.it (F.G.); 2 Istituto Stomatologico Italiano, 20122 Milan, Italy; cristian.coraini@fastwebnet.it (C.C.); andyebor@gmail.com (A.E.B.); 3 Department of Neurosciences, Reproductive Sciences and Odontostomatological Sciences, University of Naples Federico II, 80131 Naples, Italy

**Keywords:** zygomatic implants, severe maxillary atrophy, maxillary rehabilitation, treatment hierarchy, patient selection, overtreatment, patient-reported outcomes, implant dentistry, narrative review

## Abstract

*Background and Objectives:* Zygomatic implants have progressively become a widely adopted graft-free treatment option for the rehabilitation of severe maxillary atrophy. However, the expansion of their clinical indications has generated debate regarding patient selection, treatment hierarchy, and potential overtreatment. This review aimed to critically analyze current evidence regarding the use of zygomatic implants in the rehabilitation of the atrophic maxilla. *Methods:* A comprehensive narrative review of the literature was conducted through searches of PubMed/MEDLINE, Scopus, Web of Science, and the Cochrane Library. Relevant clinical studies, systematic and narrative reviews, and consensus statements published in English up to 27 March 2026 were critically analyzed to provide a comprehensive narrative synthesis focusing on treatment indications, surgical protocols, complications, patient-reported outcomes, and evidence gaps. *Results:* The reviewed literature consistently reported high implant survival rates and highlighted the role of zygomatic implants in reducing the need for extensive grafting procedures in selected patients with severe maxillary atrophy. The most frequently reported complications included sinus-related disorders, soft-tissue complications, prosthetic problems, and challenges associated with the management of implant failure. Different surgical approaches, including intra-sinus, sinus-slot, and extra-sinus techniques, were described. However, substantial heterogeneity emerged regarding treatment algorithms, complication reporting, success criteria, and patient-reported outcome assessment. *Conclusions:* Zygomatic implants represent an established treatment option associated with high reported implant survival in selected patients with severe maxillary atrophy, particularly when conventional implant placement would require extensive reconstructive procedures. However, in borderline clinical situations characterized by partial residual bone availability, treatment planning should also consider less invasive alternatives, including short implants, tilted implants, regenerative procedures, or customized subperiosteal implants, according to individual anatomical, prosthetic, and patient-related factors. Greater standardization of indications, complication reporting, and patient-reported outcome assessment is needed to better define the role of zygomatic implants within evidence-based treatment planning for the atrophic maxilla.

## 1. Introduction

The progressive increase in life expectancy and the aging of the global population have contributed to a growing prevalence of edentulism worldwide [1]. Despite significant advances in preventive dentistry and oral rehabilitation, complete or partial tooth loss still represents a major public health issue, particularly among elderly individuals [2]. Edentulism is associated with impaired mastication, altered phonetics, aesthetic concerns, nutritional deficiencies, and reduced quality of life, often affecting social interactions and psychological well-being [3]. Implant-supported prosthetic rehabilitation is widely regarded as a highly predictable treatment option for many edentulous patients because of its documented benefits in terms of function, comfort, and patient satisfaction. However, treatment selection should be individualized according to the patient’s systemic health, anatomical conditions, functional needs, and economic considerations [4,5].

However, the rehabilitation of patients presenting with advanced maxillary resorption remains one of the most challenging conditions in implant dentistry and oral surgery [6]. Following tooth loss, the maxillary alveolar process undergoes progressive centripetal and vertical bone resorption, frequently associated with maxillary sinus pneumatization and reduced bone density [7]. This phenomenon may be further aggravated by long-term use of removable prostheses and by age-related bone remodeling processes. As a consequence, the residual bone volume available for implant placement may become insufficient for conventional implant-supported rehabilitation, particularly in the posterior maxilla [8].

Several treatment approaches have been proposed over the years for the management of severely atrophic maxillae [9]. These include guided bone regeneration, autologous bone grafts harvested from intraoral or extraoral donor sites, Le Fort I osteotomy with interpositional grafting, maxillary sinus floor elevation procedures, distraction osteogenesis, and the use of short or tilted implants without regenerative techniques [10,11]. Although these procedures may provide predictable clinical outcomes in selected cases, they are often associated with increased morbidity, prolonged treatment duration, higher biological and economic costs, and considerable operator-dependent variability. In addition, advanced reconstructive procedures may require multiple surgical stages and extended healing periods before definitive prosthetic rehabilitation can be completed [12].

In this context, zygomatic implants were introduced by Brånemark as a graft-free alternative for the rehabilitation of patients with severe maxillary resorption and maxillary defects following tumor resection [13]. Unlike conventional implants, zygomatic implants achieve anchorage in the zygomatic bone, allowing the clinician to bypass the compromised maxillary anatomy and obtain high primary stability even in the absence of adequate residual bone volume [14]. This biomechanical concept enabled the development of immediate or early loading protocols and significantly reduced the need for extensive bone augmentation procedures [15].

Since their introduction, zygomatic implants have gained increasing popularity, and numerous clinical studies have reported high survival rates and favorable long-term outcomes [16]. As surgical experience has expanded, the indications for zygomatic implants have progressively evolved from a rescue therapy for extremely compromised maxillae to a widely adopted treatment option in the rehabilitation of severely atrophic maxillae. At the same time, substantial technological progress has transformed the planning and execution of zygomatic surgery. The introduction of cone beam computed tomography (CBCT), digital prosthetic planning, guided surgery, and dynamic navigation systems has improved surgical precision and facilitated prosthetically driven treatment approaches [17].

Parallel to these technological advances, several modifications of the original surgical technique have been proposed [18]. While the traditional Brånemark approach involved an intra-sinus implant trajectory, alternative techniques, such as the sinus-slot and extra-sinus approaches, were later introduced in an attempt to improve prosthetic emergence profiles, reduce sinus-related complications, and simplify prosthetic management [19]. Additional refinements in surgical protocols and implant positioning strategies have further contributed to expanding the indications for zygomatic rehabilitation in contemporary implant dentistry [20,21,22].

Despite these advances, the increasing use of zygomatic implants has generated considerable debate regarding their proper indications and the potential risk of overutilization. Although zygomatic implants may reduce treatment time and avoid extensive regenerative procedures, they are associated with specific biological and technical complications, including sinusitis, soft-tissue dehiscence, oroantral communication, prosthetic complications, and difficulties in the management of implant failure [15,23,24]. Furthermore, zygomatic implant surgery remains technically demanding because of the anatomical complexity of the maxillary and zygomatic regions, the proximity to critical anatomical structures, and the steep surgical learning curve associated with the procedure.

In this regard, recent consensus statements and expert reviews have emphasized the importance of adequate training, surgical experience, and careful patient selection in reducing complications and improving treatment outcomes [25,26]. At the same time, increasing attention has been directed toward the concept of minimally invasive therapy. Recent consensus recommendations suggest that zygomatic implants should not automatically replace conventional reconstructive options in cases where sufficient residual bone is still available for short implants, tilted implants, or regenerative procedures [27]. Consequently, the decision-making process in patients with severely atrophic maxilla has become increasingly complex and multifactorial.

Indeed, treatment planning is not determined exclusively by anatomical limitations. Patient expectations, systemic conditions, economic considerations, clinician preference, treatment time, and prosthetic requirements may all influence the therapeutic decision-making process. As a result, treatment selection may not always rely solely on evidence-based criteria but may also reflect subjective and context-dependent variables. This has raised concerns regarding the possibility that zygomatic implants may occasionally be proposed in clinical situations where less invasive reconstructive alternatives could still provide satisfactory outcomes.

Although the scientific literature on zygomatic implants has grown substantially over the last two decades, universally accepted evidence-based guidelines regarding their indications are still lacking. Current recommendations are frequently based on expert opinion and consensus reports rather than on high-level comparative evidence. Moreover, there is still a limited number of randomized controlled trials directly comparing zygomatic implants with alternative reconstructive approaches in borderline clinical scenarios [26]. Another important limitation concerns the heterogeneity of outcome assessment. While implant survival rates are consistently reported, less attention has been directed toward patient-reported outcome measures (PROMs), prosthetic complications, sinus health, and long-term maintenance. Taken together, these limitations highlight the absence of a comprehensive synthesis integrating current evidence with clinical decision-making considerations, including treatment hierarchy, patient selection, potential overtreatment, consensus recommendations, and existing evidence gaps in zygomatic implant rehabilitation. Given the predominance of retrospective studies, expert consensus statements, narrative reviews, and heterogeneous outcome measures in the available literature, a comprehensive narrative review was considered the most appropriate approach to critically synthesize current concepts, controversies, and evidence gaps regarding the use of zygomatic implants in the atrophic maxilla.

Therefore, the aim of this comprehensive review is to critically analyze current recommendations regarding the use of zygomatic implants in the rehabilitation of the atrophic maxilla, with particular attention to treatment indications, consensus statements, treatment hierarchy, and potential gaps between available evidence and clinical practice. Specifically, this review aims to: (1) summarize current treatment-planning concepts and decision-making frameworks for the rehabilitation of the atrophic maxilla; (2) critically discuss the available evidence regarding clinical outcomes, complications, and patient-reported outcomes associated with zygomatic implants; and (3) identify clinical scenarios in which the risk of overtreatment may be greatest and where further comparative evidence is needed. Clarifying the boundaries between appropriate indications and potential overutilization may contribute to a more standardized, patient-centered, and biologically driven approach to the rehabilitation of the atrophic maxilla.

## 2. Materials and Methods

### 2.1. Literature Search Strategy

A literature search was conducted in PubMed/MEDLINE, Scopus, Web of Science, and the Cochrane Library to identify relevant publications addressing the use of zygomatic implants in the rehabilitation of the atrophic maxilla. The search was designed to support a comprehensive narrative synthesis of the available evidence, with particular attention to clinical indications, treatment planning, surgical approaches, complications, consensus recommendations, patient-reported outcomes, prosthetic implications, and existing evidence gaps.

Articles published in English up to 27 March 2026 were considered. No restriction regarding publication year was applied. The electronic search was performed using combinations of Medical Subject Headings (MeSH) terms and free-text keywords related to zygomatic implants and maxillary atrophy. The following search strategy was used:

((“zygomatic implant*” OR “zygoma implant*” OR “quad zygoma”) AND (“atrophic maxilla” OR “edentulous maxilla”) AND (indication* OR guideline* OR consensus OR recommendation*)).

Additional relevant publications were identified through manual examination of the reference lists of selected articles, reviews, and consensus reports. The retrieved literature was critically analyzed according to its relevance to the objectives of the present narrative review. When multiple publications addressed similar topics, preference was given to the most recent, comprehensive, and methodologically robust sources, while additional studies were considered when they provided complementary information relevant to the objectives of the review. Given the narrative nature of the manuscript and the heterogeneity of the available evidence, no formal systematic review process, PRISMA flow diagram, risk-of-bias assessment, or quantitative meta-analysis was performed.

### 2.2. Scope of the Narrative Synthesis

The narrative synthesis focused on publications addressing at least one of the following topics: clinical indications for zygomatic implants; treatment planning in severely atrophic maxillae; consensus statements or clinical recommendations; comparisons with alternative reconstructive approaches; surgical techniques; prosthetic considerations; biological and technical complications; success criteria; and outcome assessment.

Attention was given to studies and expert recommendations discussing areas of controversy, limitations of current evidence, differences among clinical recommendations, and the potential risk of overtreatment in borderline clinical scenarios. Publications with limited relevance to the rehabilitation of the atrophic maxilla or not available in English were not considered in the narrative discussion.

### 2.3. Qualitative Synthesis

The selected literature was discussed qualitatively and organized thematically according to the main objectives of the review. Attention was given to the level and nature of the available evidence, including systematic reviews, consensus statements, prospective studies, retrospective studies, and case series. However, no formal quality assessment or risk-of-bias evaluation was performed, consistent with the narrative nature of the review. The synthesis focused on indications for zygomatic implant therapy, patient selection criteria, surgical and prosthetic protocols, reported biological and technical complications, consensus recommendations, areas of controversy, and evidence gaps within the current literature.

Due to the heterogeneity of the available publications and the narrative aim of the review, findings were organized through a thematic qualitative synthesis rather than through formal systematic data extraction. In addition to evaluating the available evidence, the review aimed to critically examine inconsistencies among published recommendations and the potential risk of overtreatment in borderline clinical scenarios where alternative reconstructive approaches may still represent viable treatment options. Emphasis was placed on the relationship between current evidence, expert consensus statements, clinical decision-making, and patient-centered treatment planning.

## 3. Results

### 3.1. Residual Bone Availability and Treatment Indications in the Atrophic Maxilla

The reviewed literature consistently identifies severe maxillary atrophy as one of the principal indications for zygomatic implant rehabilitation [16]. Following tooth loss, the maxillary alveolar ridge undergoes progressive centripetal and vertical resorption associated with sinus pneumatization and reduction in bone density, resulting in a progressive decrease in the residual bone volume available for conventional implant placement [28]. Several authors additionally reported that this process may be aggravated by long-term use of removable prostheses and age-related bone remodeling changes.

The anatomical changes associated with edentulism frequently affect both the anterior and posterior maxilla [29]. In advanced cases, the combination of alveolar ridge resorption and sinus expansion may significantly compromise the possibility of obtaining adequate primary stability with conventional implants [30]. The reviewed studies described the posterior maxilla as one of the most critical anatomical areas because of limited residual bone height and poor bone quality [31].

Over time, the concept of “adequate residual bone” progressively evolved within the literature. Earlier reconstructive approaches mainly focused on the absolute quantity of residual alveolar bone, whereas more recent publications emphasized the importance of the anatomical distribution of the available bone and the possibility of exploiting extra-alveolar anchorage sites [20]. In this context, the Bedrossian classification became one of the most widely adopted systems for treatment planning in severely atrophic maxillae [32,33].

According to this classification, the maxilla is divided into four anatomical zones: zone I (premaxilla), zone II (premolar region), zone III (molar region), and zone IV (zygoma). The reviewed literature described this classification as a useful approach for evaluating residual bone distribution and selecting the most appropriate rehabilitative strategy according to the anatomical characteristics of each patient. Several studies reported that patients presenting with residual bone preservation in the anterior maxilla may still be rehabilitated using conventional implants, short implants, or tilted implants, with or without regenerative procedures [11,34]. In general, preservation of residual bone in the anterior maxilla may still allow rehabilitation through conventional implants, short implants, tilted implants, or combined treatment approaches. Conversely, extensive loss of both anterior and posterior maxillary support may require alternative anchorage strategies, including zygomatic implant rehabilitation. In these situations, the zygomatic bone was repeatedly described as a reliable anchorage site because of its cortical density and biomechanical stability [21].

Different reconstructive approaches for the rehabilitation of severely atrophic maxillae were identified in the reviewed literature. Several authors reported favorable outcomes with these techniques in selected clinical situations. However, many publications also described limitations associated with advanced grafting procedures, including increased morbidity, prolonged treatment duration, higher biological and economic costs, and the need for multiple surgical stages [35,36]. According to Moussa et al., some grafting procedures may require healing periods extending over several months before definitive prosthetic rehabilitation can be completed [37]. More recently, customized subperiosteal implants have emerged as an additional graftless treatment option for selected patients with severe maxillary atrophy. These patient-specific devices, manufactured through digital planning and CAD/CAM technologies, may allow full-arch rehabilitation without extensive bone grafting procedures. Preliminary clinical studies and recent reviews have reported encouraging short- and medium-term outcomes; however, the available evidence remains comparatively limited, and long-term data are still insufficient to establish definitive recommendations regarding their role within the treatment hierarchy of the atrophic maxilla [38,39].

In contrast, zygomatic implants were consistently presented as a graft-free alternative capable of reducing rehabilitation time and facilitating immediate or early loading protocols [40]. However, the reviewed literature also emphasized that zygomatic implants should be considered within a broader treatment hierarchy and that careful patient selection remains essential, particularly when less invasive reconstructive options may still be feasible [25,26,41]. Several studies additionally highlighted that zygomatic rehabilitation may reduce the need for extensive augmentation procedures in patients presenting with severe maxillary resorption [42]. The principal treatment options currently available for the rehabilitation of the atrophic maxilla, together with their typical indications and main characteristics, are summarized in Table 1 and may assist clinicians in selecting the most appropriate treatment strategy according to residual bone availability and patient-specific factors.

The reviewed literature also demonstrated increasing interest in treatment algorithms aimed at selecting the most appropriate rehabilitative strategy according to the degree of maxillary atrophy. Several consensus reports and expert recommendations emphasized the importance of individualized treatment planning based on residual bone anatomy, sinus configuration, prosthetic requirements, patient-related factors, and clinician experience [25,26,42].

Despite the broad acceptance of these treatment-planning concepts, important differences remain regarding the threshold at which zygomatic implants should be preferred over alternative reconstructive approaches. Current recommendations are largely based on expert consensus, retrospective studies, and clinical experience rather than on high-quality comparative evidence. Consequently, the optimal positioning of zygomatic implants within the treatment hierarchy of the severely atrophic maxilla remains an area of ongoing debate. Current evidence supports a patient-specific approach rather than the routine adoption of zygomatic implants as a universal treatment solution. Concerns regarding potential overtreatment appear to be particularly relevant in borderline clinical situations characterized by partial residual bone availability, where less invasive alternatives, such as short implants, tilted implants, regenerative procedures, or customized subperiosteal implants, may still represent viable treatment options. However, the boundaries between appropriate indications and potential overutilization remain incompletely defined because current recommendations are largely based on expert consensus rather than on high-quality comparative evidence.

### 3.2. Surgical Technique and Evolution of Zygomatic Implant Protocols

The traditional intra-sinus technique involved implant placement through the maxillary sinus with anchorage in the zygomatic bone and was initially proposed for the rehabilitation of patients presenting with severe maxillary defects or severely atrophic maxillae [13]. The original Brånemark protocol was based on a long implant trajectory extending from the residual alveolar crest to the body of the zygomatic bone [13]. The implant body passed through the maxillary sinus, while the implant head generally emerged at the level of the palatal aspect of the residual alveolar ridge. Early reports described this approach as a graft-free alternative capable of obtaining high primary stability in patients with insufficient residual maxillary bone.

Several subsequent studies focused on modifications of the original intra-sinus approach to improve prosthetic emergence, simplify prosthetic rehabilitation, and reduce sinus-related complications. Among these modifications, the sinus-slot technique proposed by Stella and Warner represented one of the first alternatives to the original Brånemark protocol [43]. This technique involved the preparation of a lateral slot along the maxillary sinus wall in order to guide implant positioning and reduce the extent of the implant trajectory within the sinus cavity [43].

Later, extra-sinus approaches became increasingly described within the literature [44]. In these protocols, the implant body was positioned external to the sinus cavity along the lateral wall of the maxilla before engaging the zygomatic bone. Several authors reported that extra-sinus techniques may facilitate prosthetic emergence closer to the alveolar crest and improve prosthetic hygiene access, particularly in patients presenting with severe buccal concavity of the maxillary wall [45,46].

The reviewed studies also highlighted that the choice between intra-sinus, sinus-slot, and extra-sinus approaches was frequently influenced by anatomical characteristics, including sinus morphology, residual alveolar bone volume, degree of maxillary concavity, and prosthetic requirements [47]. In addition, several publications reported variability in implant trajectory, implant head emergence, and extent of sinus involvement among the different techniques. The principal characteristics, potential advantages, and limitations of the main surgical approaches described in the literature are summarized in Table 2.

Different implant configurations for the rehabilitation of severely atrophic maxillae were additionally described in the literature. These included unilateral and bilateral zygomatic implants combined with conventional anterior implants, hybrid configurations with tilted implants, and quad zygoma protocols in patients presenting with extensive anterior and posterior maxillary atrophy [48]. The reviewed literature most frequently described hybrid rehabilitations combining zygomatic implants with conventional anterior implants, particularly in patients with residual anterior maxillary bone support. In contrast, quad zygoma configurations were generally reported in cases of extensive anterior and posterior maxillary atrophy characterized by the absence of sufficient residual bone for conventional implant placement.

Several studies additionally focused on the biomechanical principles underlying zygomatic implant stability. According to Bedrossian et al., successful anchorage is achieved through engagement of multiple cortical structures, including the alveolar crest, sinus floor, and zygomatic cortical bone [21]. This concept of multi-cortical anchorage was consistently associated with the high primary stability reported in zygomatic implant rehabilitation.

Immediate loading protocols represented another recurrent finding in the reviewed literature [49]. Several authors associated the predictability of immediate loading with the high insertion torque and primary stability obtained through engagement of the zygomatic cortical bone [50]. Immediate full-arch fixed rehabilitations supported by zygomatic implants were commonly described in both retrospective and prospective clinical investigations.

**Figure 1:**
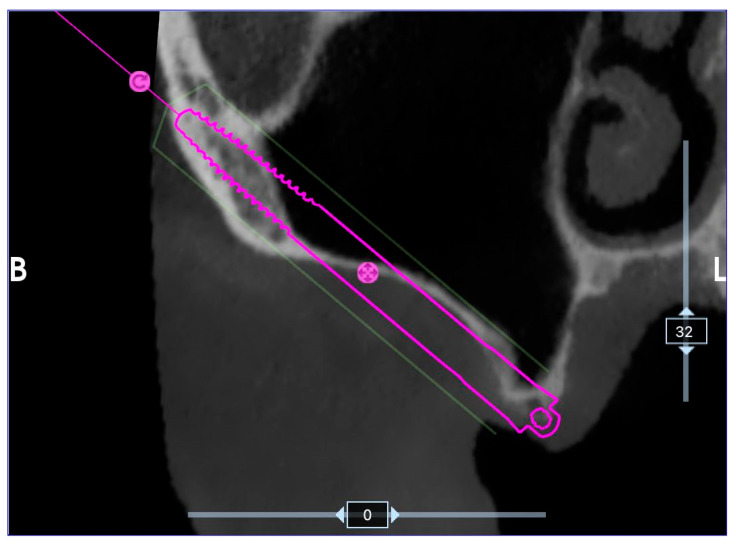
Representative CBCT-based digital planning of a zygomatic implant in a severely atrophic maxilla. The image illustrates virtual implant positioning, anatomical evaluation of the maxillary sinus and zygomatic bone, and prosthetically driven treatment planning aimed at optimizing implant trajectory and prosthetic emergence.

The integration of digital technologies into zygomatic implant surgery was also frequently reported. Cone beam computed tomography (CBCT), virtual planning software, computer-guided surgery, and dynamic navigation systems were increasingly incorporated into preoperative planning and surgical execution [42]. These technologies were primarily described as tools for improving surgical accuracy, optimizing implant trajectory, and facilitating prosthetically driven rehabilitation (Figure 1). Beyond improving implant positioning accuracy, digital planning and navigation systems have also been proposed as tools to enhance control of implant trajectory, prosthetic emergence profile, and anatomical risk management [51,52]. However, the currently available evidence remains insufficient to demonstrate a consistent reduction in complication rates when compared with conventional freehand approaches, and further comparative studies are required.

Several publications additionally emphasized the importance of accurate three-dimensional radiographic assessment because of the anatomical complexity of the zygomatic-maxillary region. The reviewed studies frequently described the proximity of critical anatomical structures, including the orbit, infraorbital nerve, infratemporal fossa, and maxillary sinus. Consequently, many authors highlighted the importance of detailed anatomical evaluation before surgery.

Surgical training and operator experience were repeatedly identified as important variables influencing treatment outcomes and complication rates [53]. The literature consistently described zygomatic implant placement as a technically demanding procedure characterized by a steep learning curve, significant anatomical variability, and high operator dependence. Although several surgical approaches have been described and successfully applied in clinical practice, current evidence does not clearly demonstrate the superiority of one technique over another. Technique selection therefore remains largely dependent on patient-specific anatomical characteristics, prosthetic requirements, and surgeon experience. The absence of standardized comparative studies limits definitive conclusions regarding the optimal surgical approach.

### 3.3. Clinical Outcomes, Complications, and Outcome Assessment

Most of the reviewed studies reported high survival rates for zygomatic implants during both medium- and long-term follow-up periods. According to the systematic review by Candel-Martí et al., as well as Chrcanovic et al., survival rates frequently exceeded 95%, with several retrospective and prospective investigations reporting favorable clinical outcomes after immediate or delayed loading protocols [46,54]. Similar findings were described in subsequent clinical studies and consensus reports evaluating zygomatic rehabilitation in patients with severe maxillary atrophy [28]. The literature consistently identified high primary stability as one of the main characteristics of zygomatic implants. Several authors have associated this feature with the dense cortical structure of the zygomatic bone and the concept of multi-cortical anchorage involving the alveolar crest, sinus floor, and zygomatic cortical bone [21]. Because of these biomechanical characteristics, immediate or early loading protocols were frequently reported among the included studies [15].

In addition to implant survival, several publications evaluated prosthetic rehabilitation outcomes. Most studies described high prosthetic survival rates and restoration stability during follow-up periods. Full-arch fixed rehabilitations supported by zygomatic implants were commonly associated with rapid restoration of function and reduced treatment time when compared with staged grafting procedures [40]. Despite these favorable outcomes, the reviewed literature also documented a broad spectrum of biological and technical complications associated with zygomatic implant therapy. Among biological complications, sinusitis represented one of the most frequently reported adverse events [54]. Other reported complications included soft-tissue dehiscence, mucosal inflammation, oroantral communication, peri-implant infection, paresthesia, and local discomfort [55]. Some studies additionally described difficulties associated with the management and removal of failed zygomatic implants because of the anatomical complexity of the procedure.

The prevalence of sinus-related complications varied considerably among studies. Several publications highlighted the absence of universally accepted diagnostic criteria for postoperative sinusitis, contributing to substantial heterogeneity in reported incidence rates. Furthermore, differences in surgical technique, implant trajectory, follow-up duration, and radiographic assessment methods complicated direct comparison between investigations.

The reviewed literature also reported technical and prosthetic complications related to zygomatic rehabilitation [56]. These mainly included prosthetic screw loosening, prosthetic fractures, hygiene difficulties, and complications associated with prosthetic emergence profile management, particularly in cases involving extra-sinus implant positioning. Some authors emphasized that prosthetic emergence may influence phonetics, oral hygiene maintenance, and patient comfort.

Several studies evaluated the influence of surgical technique on complication rates and prosthetic outcomes. While intra-sinus protocols were historically associated with a greater implant trajectory within the sinus cavity, extra-sinus approaches were introduced to improve prosthetic emergence and potentially reduce sinus-related complications [19]. Although direct comparisons remain limited, several authors have suggested that intra-sinus approaches may be more frequently associated with sinus-related complications because of the greater involvement of the maxillary sinus, whereas extra-sinus techniques may be associated with a higher prevalence of soft-tissue complications, hygiene difficulties, and prosthetic maintenance challenges. However, these observations should be interpreted cautiously because of substantial heterogeneity in study design, patient selection, and complication reporting [19,28,52]. However, the reviewed studies demonstrated variability in complication reporting and did not provide uniform conclusions regarding the superiority of one technique over another. Interpretation of the available evidence is further complicated by the substantial heterogeneity in outcome assessment across studies. While implant survival is commonly reported, the definitions and methods used to evaluate complications, prosthetic outcomes, sinus health, and patient-reported outcomes vary considerably, limiting direct comparison among studies and contributing to uncertainty regarding the overall effectiveness of zygomatic implant rehabilitation.

Another recurrent finding in the literature concerned the importance of operator-related factors. Zygomatic implant placement was consistently described as a technically demanding procedure characterized by a steep learning curve and substantial anatomical complexity [25,42]. Several authors identified clinician experience, anatomical knowledge, and accurate preoperative planning as fundamental factors influencing surgical safety and long-term treatment outcomes.

The methods used to assess treatment success were found to be heterogeneous among studies. Most investigations primarily focused on implant survival rates, whereas fewer studies evaluated broader outcome parameters, such as prosthetic maintenance, sinus health, patient satisfaction, aesthetics, phonetics, and oral-health-related quality of life. The predominance of implant survival as the principal reported outcome may limit the ability to fully assess the long-term clinical risk profile of zygomatic implant rehabilitation. Implant survival alone does not necessarily reflect sinus health, prosthetic maintenance requirements, soft-tissue stability, patient satisfaction, or overall treatment burden. Future studies should therefore adopt more comprehensive outcome measures integrating implant survival, sinus health, prosthetic performance, maintenance requirements, and patient-reported outcomes in order to provide a more clinically meaningful assessment of treatment success. In this context, Aparicio et al. proposed the Zygomatic Success Code (ZSC), a multidimensional classification system incorporating surgical, prosthetic, biological, and patient-related parameters [20].

Several research additionally highlighted the limited evaluation of patient-reported outcome measures (PROMs) in zygomatic implant rehabilitation. Although many studies described favorable patient satisfaction following treatment, standardized assessment tools and uniform outcome measures were inconsistently applied across the available literature.

### 3.4. Consensus Statements and Evidence Gaps

Recent consensus reports and expert recommendations increasingly attempted to define treatment algorithms and clinical indications for zygomatic implant rehabilitation. The ITI consensus report emphasized the importance of comprehensive preoperative assessment, prosthetically driven planning, adequate surgical training, and detailed radiographic evaluation through cone beam computed tomography [25]. Similar recommendations were proposed by Pala et al., who highlighted the relevance of careful patient selection and standardized surgical planning in reducing complications and improving treatment predictability [26]. In addition, several consensus initiatives additionally focused on the role of minimally invasive treatment strategies in the management of advanced maxillary resorption. Short implants and alternative reconstructive approaches should be considered whenever sufficient residual bone volume remains available for conventional implant-supported rehabilitation [26]. Within these recommendations, zygomatic implants were primarily indicated in cases characterized by severely atrophic maxilla and insufficient residual bone for conventional implant placement.

The reviewed literature demonstrated substantial heterogeneity regarding treatment indications and decision-making criteria. Different studies proposed variable thresholds of residual bone volume for selecting between grafting procedures, short implants, tilted implants, and zygomatic implants. Several authors also emphasized that treatment planning may be influenced by additional factors, including sinus anatomy, prosthetic requirements, patient expectations, systemic conditions, treatment time, economic considerations, and clinician experience.

Considerable variability was additionally observed among surgical protocols and implant positioning strategies. The reviewed studies included intra-sinus, sinus-slot, and extra-sinus approaches, frequently combined with different prosthetic loading protocols and implant configurations. Variability was also found in relation to the use of digital planning systems, guided surgery, and navigation-assisted procedures.

The available literature further demonstrated marked heterogeneity in the definition and reporting of complications. While some studies primarily focused on implant survival, others additionally evaluated sinus health, prosthetic complications, soft-tissue conditions, or patient satisfaction. The absence of standardized outcome measures limited direct comparison between studies and complicated interpretation of the available evidence. Randomized controlled trials directly comparing zygomatic implants with alternative reconstructive approaches were found to be limited. Most of the currently available evidence consisted of retrospective analyses, case series, narrative reviews, and expert consensus reports. According to Pala et al., several current recommendations are therefore still largely based on expert opinion rather than on high-level comparative evidence [26]. The strength of the available evidence varies considerably according to the specific clinical question. Evidence supporting the use of zygomatic implants in patients with severe maxillary atrophy is primarily derived from systematic reviews, retrospective cohort studies, and consensus reports demonstrating favorable implant survival outcomes. In contrast, recommendations regarding treatment selection in borderline clinical scenarios, including the choice between zygomatic implants and less invasive alternatives, remain largely based on expert opinion, consensus statements, and clinical experience because of the limited availability of high-quality comparative studies. Another recurring finding concerned the limited availability of long-term data evaluating biological complications and maintenance requirements. Although implant survival was frequently reported, few studies investigated long-term sinus health, prosthetic maintenance burden, peri-implant soft-tissue stability, and the management of failed zygomatic implants.

The reviewed literature also revealed limited and heterogeneous evaluation of patient-reported outcome measures (PROMs). While several studies described favorable patient satisfaction and rapid functional rehabilitation following zygomatic implant therapy, standardized tools for assessing comfort, phonetics, aesthetics, quality of life, and prosthetic satisfaction were inconsistently applied [26].

Overall, the available evidence demonstrated increasing clinical interest in zygomatic implant rehabilitation together with progressive efforts toward treatment standardization. However, important evidence gaps remained regarding patient selection criteria, comparative effectiveness against alternative reconstructive approaches, standardized outcome assessment, and long-term patient-centered evaluation. Several clinically relevant questions also remain insufficiently addressed by the current literature, including the long-term incidence and management of chronic sinus complications, the development of standardized protocols for the treatment of failed zygomatic implants, the influence of operator experience and learning curve on treatment outcomes, and the identification of evidence-based criteria for treatment selection in borderline clinical situations. Addressing these issues will require prospective comparative studies and more standardized outcome assessment methods.

## 4. Discussion

The rehabilitation of the severely atrophic maxilla remains a complex clinical scenario in which anatomical limitations, prosthetic requirements, patient-related factors, and surgical expertise must be considered together [57]. The available literature suggests that zygomatic implants have progressively moved from a rescue option for extreme maxillary atrophy or post-resective defects to a more frequently adopted treatment modality in contemporary implant dentistry [58]. This evolution reflects the high survival rates reported in the literature, the possibility of avoiding extensive grafting procedures, and the increasing availability of digital planning and prosthetically driven workflows [14,25]. However, interpretation of the available evidence requires caution. Much of the current literature consists of retrospective cohort studies, case series, narrative reviews, and expert consensus reports, while high-quality prospective comparative studies remain limited. Consequently, many recommendations regarding indications, treatment hierarchy, and clinical decision-making continue to rely on relatively low levels of evidence and should be interpreted within the methodological limitations of the existing literature. However, the same expansion of indications also raises important questions regarding treatment hierarchy, appropriate patient selection, and the potential risk of overtreatment.

A first relevant aspect concerns the definition of “adequate bone” in the atrophic maxilla. Historically, the indication for advanced reconstructive procedures was mainly based on the amount of residual alveolar bone [59]. More recent approaches, however, have shifted attention toward the anatomical distribution of the remaining bone and its potential role in strategic implant anchorage. In this regard, the Bedrossian classification has contributed to reframing treatment planning by dividing the maxilla into different anatomical zones and linking residual bone distribution to specific rehabilitative options [21,28]. This concept is clinically important because it suggests that bone availability should not be interpreted only as a linear measurement of height or width, but as a three-dimensional condition that must be evaluated in relation to the planned prosthetic design and implant configuration.

Within this framework, zygomatic implants represent a logical solution when the residual alveolar bone is insufficient to support conventional implants and when grafting procedures would significantly increase treatment time, morbidity, or complexity. Their main advantage is not merely the use of an alternative anchorage site, but the possibility of changing the reconstructive strategy from bone-dependent rehabilitation to anatomy-guided anchorage. This is particularly relevant in patients with extensive posterior maxillary resorption, severe sinus pneumatization, or generalized atrophy involving both anterior and posterior regions [60]. Nevertheless, the literature also indicates that not all atrophic maxillae require zygomatic implants [61]. When residual bone is still available, short implants, tilted implants, sinus augmentation, or guided bone regeneration may remain valid alternatives, depending on the anatomical and prosthetic scenario [26,62].

This point is central to the debate on overtreatment. The possibility of providing an immediate or graftless fixed rehabilitation may lead clinicians to favor zygomatic implants even in borderline cases where less invasive options could still be feasible [63]. From a biological perspective, the least invasive yet effective treatment should generally be preferred, particularly when comparable functional outcomes may be achieved with conventional or regenerative approaches. The indication for zygomatic implants should therefore not be based only on technical feasibility, but on a balanced evaluation of invasiveness, expected benefit, complication profile, maintenance requirements, and patient preference. Examples of potentially controversial clinical situations include patients presenting with partial residual anterior maxillary bone support, in whom rehabilitation may still be feasible through short implants, tilted implants, or limited regenerative procedures despite the technical feasibility of zygomatic implant placement. In these cases, treatment selection should be guided by a comprehensive evaluation of anatomical conditions, systemic health, treatment duration, surgical morbidity, prosthetic requirements, and patient preferences rather than by implant feasibility alone. In this sense, zygomatic implantology should not be interpreted as a shortcut that replaces reconstructive decision-making, but as one component of a broader treatment algorithm. It should be emphasized that concerns regarding potential overtreatment are currently supported primarily by expert opinion, consensus recommendations, and the principles of minimally invasive treatment planning rather than by high-level comparative evidence. Direct prospective studies comparing zygomatic implants with alternative reconstructive strategies in borderline clinical scenarios remain limited. Consequently, the debate surrounding overtreatment reflects an important clinical and ethical consideration, but one that still requires stronger comparative evidence to be definitively validated.

The comparison between zygomatic implants and graft-based reconstruction remains difficult because the available literature is heterogeneous and often lacks direct comparative evidence. Bone augmentation procedures, sinus floor elevation, and staged grafting protocols may provide predictable outcomes in selected patients, but they are frequently associated with longer rehabilitation times, increased morbidity, and delayed loading [9]. Conversely, zygomatic implants may reduce treatment duration and avoid donor-site morbidity, but they introduce a different risk profile, including sinus-related complications, soft-tissue dehiscence, prosthetic emergence challenges, and complex management in case of implant failure [54]. Therefore, the clinical question should not be whether zygomatic implants are superior to grafting in general, but in which anatomical and patient-related conditions they provide a more appropriate risk-benefit balance.

The evolution of surgical techniques further complicates this assessment. The original intra-sinus approach described by Brånemark has been progressively modified through sinus-slot and extra-sinus protocols, with the aim of improving prosthetic emergence, reducing sinus involvement, and adapting implant positioning to individual anatomy [13,19]. These modifications have broadened the technical possibilities of zygomatic rehabilitation, but they have also introduced variability in surgical execution and outcome reporting. Consequently, comparisons among studies remain challenging and contribute to the heterogeneity of the available evidence. For instance, extra-sinus trajectories may improve prosthetic access and emergence profile in selected anatomies, but they may also increase the exposure of the implant body to soft-tissue complications if keratinized tissue support and prosthetic design are inadequate [64]. This reinforces the need to evaluate surgical technique not as an isolated variable, but as part of an integrated surgical-prosthetic plan.

The prosthetic dimension is particularly relevant. In zygomatic implant rehabilitation, implant survival alone does not necessarily correspond to treatment success. A surviving implant may still be associated with prosthetic difficulties, hygiene limitations, phonetic issues, soft tissue inflammation, or patient dissatisfaction [65]. This is one of the main weaknesses of the current literature: survival rates are frequently reported, while broader measures of success are less consistently assessed. The Zygomatic Success Code proposed by Aparicio et al. represents an important attempt to move beyond survival-based reporting by incorporating surgical, biological, prosthetic, and patient-related criteria [66]. However, its limited adoption in clinical studies reduces the comparability of outcomes and prevents a more mature interpretation of treatment success.

Sinus health is another key issue. Sinusitis is among the most frequently reported complications after zygomatic implant placement, but its real incidence remains difficult to interpret because diagnostic criteria vary widely among studies [54]. In fact, the discrepancy between radiological findings and clinical symptomatology reported in the literature suggests that sinus alterations may often remain subclinical or asymptomatic, potentially leading to an underestimation of sinus involvement [67]. Some reports rely on clinical symptoms, others include radiographic findings, and others do not clearly distinguish transient sinus inflammation from chronic sinus disease. This lack of standardization may either underestimate or overestimate the clinical relevance of sinus complications. Future studies should define sinusitis using reproducible clinical and radiographic criteria and, ideally, include preoperative and postoperative otolaryngological evaluation in selected patients. Nevertheless, given the postoperative complications involving the maxillary sinus described in cases of zygomatic implant rehabilitation, it would be advisable to perform a clinical evaluation of the sinonasal tract, including a symptom-based analysis using validated clinical criteria, supplemented by radiographic examinations and an otolaryngological assessment [68].

The influence of the operator is another major element emerging from the literature. Zygomatic implant placement is anatomically demanding and differs substantially from conventional implant surgery. Small deviations in implant trajectory may affect sinus involvement, orbital safety, prosthetic emergence, and soft-tissue conditions. For this reason, surgical experience, anatomical knowledge, and accurate planning are repeatedly emphasized in consensus recommendations [25]. Current consensus recommendations also emphasize the importance of structured training pathways, supervised surgical experience, and dedicated educational programs when introducing clinicians to zygomatic implant rehabilitation. These measures are considered essential for minimizing complications and improving treatment predictability. This operator dependence may partly explain the variability in complications and outcomes reported across studies. It also suggests that the indication for zygomatic implants should take into account not only the patient’s anatomy, but also the clinician’s training and the availability of an experienced multidisciplinary team [53].

Digital workflows have the potential to reduce some of this variability, but they should not be regarded as a substitute for surgical expertise. CBCT-based planning, guided surgery, and dynamic navigation may improve visualization of anatomical structures and help optimize implant trajectory [25]. However, zygomatic implant placement remains a complex procedure in which intraoperative judgment is still essential. Inaccurate segmentation, guide instability, limited surgical access, or anatomical variability may affect the reliability of digital tools. Therefore, digital planning should be considered an adjunct to anatomical knowledge and surgical experience rather than an independent guarantee of safety.

A further consideration concerns patient-centered outcomes. Zygomatic implants are often presented as a treatment capable of rapidly restoring function, aesthetics, and quality of life. While this is clinically plausible and frequently reported in observational studies, standardized patient-reported outcome measures remain insufficiently represented in the literature [26]. This is a relevant limitation because patients may judge treatment success differently from clinicians. Future studies should incorporate validated patient-reported outcome measures (PROMs) to allow a more comprehensive assessment of treatment success from the patient’s perspective. Instruments such as the Oral Health Impact Profile (OHIP-14 and OHIP-EDENT) may provide valuable information regarding oral-health-related quality of life, while visual analogue scales (VAS) and structured satisfaction questionnaires can be used to assess comfort, aesthetics, speech, masticatory function, and overall treatment satisfaction. Given the specific prosthetic and anatomical characteristics of zygomatic implant rehabilitation, greater standardization in PROM selection and reporting would facilitate comparison among studies and improve the evaluation of patient-centered outcomes [69,70].

The present review also highlights the need for greater clarity in the treatment hierarchy of the atrophic maxilla. Current consensus statements support minimally invasive decision-making and suggest that zygomatic implants should be reserved for cases in which conventional or less invasive alternatives are not suitable or would provide a less favorable risk-benefit profile [26]. However, the boundary between appropriate indication and overextension remains insufficiently defined. This is especially true in borderline cases where short implants, tilted implants, sinus augmentation, or zygomatic implants may all be technically possible. Taken together, the available literature supports the use of zygomatic implants in appropriately selected patients; however, the current evidence remains insufficient to establish universally accepted treatment thresholds or to definitively determine the optimal position of zygomatic implants within the therapeutic hierarchy of the severely atrophic maxilla.

In such scenarios, treatment planning should be individualized through a careful evaluation of patient-related characteristics, anatomical limitations, and operative challenges associated with both local tissue volumes and systemic conditions, while actively involving the patient in the decision-making process. This personalized approach should explicitly balance biological cost, surgical morbidity, technical complexity, rehabilitation time, prosthetic prognosis, and patient expectations, taking into consideration all factors that may influence the rehabilitative pathway and long-term outcomes [55]. At the same time, treatment individualization cannot be separated from an appropriate patient selection process for prosthetic rehabilitation, with particular attention to clinical situations potentially exposed to the risk of overtreatment, always within a comprehensive risk–benefit assessment framework.

The present review also presents some inherent limitations that should be acknowledged. As a comprehensive narrative review, it was designed to provide a critical and clinically oriented synthesis of current evidence, consensus recommendations, areas of controversy, and existing knowledge gaps regarding zygomatic implant rehabilitation. Consequently, no formal systematic review methodology was adopted, and no systematic data extraction, risk-of-bias assessment, or meta-analysis was performed. Although a structured literature search was conducted to improve transparency and comprehensiveness, the narrative nature of the review may introduce a degree of interpretative subjectivity in the selection and discussion of the available evidence. In addition, only publications published in English were considered, which may have resulted in the exclusion of potentially relevant studies reported in other languages and may therefore represent a source of language bias.

First, much of the available literature consists of retrospective studies, case series, expert opinions, and consensus statements. Although these sources provide valuable clinical information, they offer a lower level of evidence than prospective comparative trials. Second, the included studies vary considerably in terms of patient selection, implant configuration, surgical technique, loading protocol, prosthetic design, follow-up duration, and complication reporting. This heterogeneity limits the possibility of drawing firm conclusions regarding the superiority of one technique or treatment strategy over another. Third, many studies focus primarily on implant survival, while reporting of prosthetic complications, sinus outcomes, soft-tissue stability, and PROMs remains inconsistent.

Another limitation concerns the lack of evidence in borderline indications. The strongest rationale for zygomatic implants exists in cases of extreme maxillary atrophy where conventional implants cannot be placed without major reconstruction. The evidence is less clear when zygomatic implants are compared with alternative reconstructive strategies in patients with partial residual bone availability. These are precisely the clinical situations in which the risk of overtreatment is highest and where stronger comparative evidence would be most useful. Well-designed prospective studies should therefore focus on these borderline scenarios rather than only reporting survival rates in severely compromised cases.

Future research should move beyond implant survival as the primary endpoint. Prospective multicenter studies should include standardized definitions of biological and prosthetic complications, uniform reporting of sinus health, validated PROMs, and longer follow-up periods. Comparative studies between zygomatic implants, short implants, tilted implants, sinus augmentation, and graft-based reconstruction would be particularly valuable for defining clearer indications. In addition, future investigations should evaluate the influence of surgical technique, digital planning, soft tissue management, prosthetic design, and operator experience on long-term outcomes. A minimum reporting framework for future clinical studies should include implant survival, standardized definitions of biological and technical complications, sinus health outcomes, validated patient-reported outcome measures, detailed descriptions of surgical and prosthetic protocols, and information regarding operator experience. Greater standardization of reporting would substantially improve the comparability and clinical applicability of future evidence.

From a clinical perspective, the future of zygomatic implantology should be directed toward standardization rather than simple expansion of indications. The procedure has clear value in selected patients with severely atrophic maxilla, but its use should remain anchored to a careful diagnostic process and to a biologically driven treatment plan. A more consistent adoption of multidimensional success criteria, such as those proposed by Aparicio et al., together with standardized consensus-based algorithms, may help reduce variability in clinical decision-making and improve comparability across studies [20].

Overall, the current literature supports zygomatic implants as an effective and predictable option for the rehabilitation of selected patients with severely atrophic maxillae. Nevertheless, their use should be considered within a structured treatment hierarchy that includes fewer invasive alternatives whenever anatomically and prosthetically appropriate. Clarifying the indications, improving outcome standardization, and integrating patient-centered measures will be essential to ensure that zygomatic implant therapy continues to develop as a predictable, safe, and evidence-based treatment modality rather than as an overextended solution for all forms of maxillary atrophy.

## 5. Conclusions

Zygomatic implants have progressively evolved from a rescue approach for extreme maxillary defects to a widely adopted treatment option for the rehabilitation of severe maxillary atrophy. Their ability to provide graft-free rehabilitation and facilitate immediate loading has contributed to the increasing adoption of this technique in contemporary implant dentistry. However, the present review highlights that the expansion of clinical indications has occurred in the context of heterogeneous protocols, variable success criteria, and limited high-level comparative evidence. Although favorable survival rates are consistently reported, the available literature still provides insufficient standardization regarding patient selection, complication assessment, long-term maintenance, and patient-centered outcomes.

The available literature suggests that zygomatic implants may represent a valuable treatment option in carefully selected patients with severe maxillary atrophy and insufficient residual bone for predictable conventional implant rehabilitation, particularly when alternative reconstructive approaches are unlikely to provide a predictable or less invasive solution. Conversely, when adequate residual bone remains available, less invasive alternatives such as conventional implants, short implants, tilted implants, or regenerative procedures should also be considered within the treatment-planning process. Clinical decision-making should always integrate anatomical conditions, sinus anatomy, systemic factors, operator experience, prosthetic requirements, and patient expectations. Comprehensive preoperative assessment, including CBCT evaluation and prosthetically driven treatment planning, should be regarded as essential components of the diagnostic workflow. Future research should focus on prospective comparative investigations, standardized outcome assessment, and greater integration of patient-reported outcome measures to better define the role of zygomatic implants within evidence-based treatment planning for the atrophic maxilla.

## Figures and Tables

**Table 1:** Main treatment options for rehabilitation of the atrophic maxilla, including their typical indications and principal characteristics.

Treatment Option	Typical Indications	Main Characteristics
**Bone grafting procedures**	Moderate-to-severe maxillary atrophy with residual bone volume suitable for reconstruction	Allows placement of conventional implants following bone augmentation; associated with longer treatment times, increased morbidity, and multiple surgical stages
**Short implants**	Moderate maxillary atrophy with sufficient residual bone height	Minimally invasive approach with reduced surgical morbidity; applicability may be limited in cases of severe maxillary atrophy
**Tilted implants**	Posterior maxillary atrophy, particularly in the presence of sinus pneumatization and residual anterior bone support	Graftless approach that may avoid sinus augmentation; requires careful prosthetic planning and biomechanical considerations
**Zygomatic implants**	Severe-to-extreme maxillary atrophy with insufficient residual bone volume for predictable conventional implant placement	Graftless rehabilitation option allowing immediate loading in selected cases; technically demanding and associated with potential sinus-related complications
**Customized subperiosteal implants**	Severe maxillary atrophy in selected patients unsuitable for conventional implant placement or extensive grafting procedures	Patient-specific CAD/CAM-designed framework supported by the residual maxillary bone; promising early outcomes but limited long-term evidence

Note: Indications are intended as general clinical scenarios derived from the available literature and should not be interpreted as absolute treatment recommendations.

**Table 2:** Main characteristics, potential advantages, and limitations of the intra-sinus, sinus-slot, and extra-sinus approaches for zygomatic implant rehabilitation.

Surgical Approach	Main Characteristics and Implant Trajectory	Potential Advantages	Main Limitations
**Intra-sinus**	Implant trajectory passes through the maxillary sinus before engaging the zygomatic bone	Long-term clinical experience; high primary stability; extensive clinical documentation	Greater sinus involvement; possible palatal emergence; more challenging hygiene maintenance
**Sinus-slot**	Implant guided through a lateral slot prepared along the sinus wall, reducing the intra-sinus trajectory	Improved visualization and trajectory control; reduced intra-sinus involvement compared with the traditional intra-sinus approach	Technique-sensitive; sinus manipulation may still be required
**Extra-sinus**	Implant positioned external to the sinus cavity along the lateral maxillary wall before engaging the zygomatic bone; emergence generally closer to the alveolar crest	Improved prosthetic emergence profile; easier hygiene access; reduced sinus involvement	Greater dependence on soft-tissue management; potential soft-tissue complications if prosthetic conditions are unfavorable

## Data Availability

No new data were created or analyzed in this study. Data sharing is not applicable to this article.
